# Trajectories of change in mothers’ parenting confidence and relationship with baby: a 15-month qualitative longitudinal study

**DOI:** 10.1186/s12884-025-07683-0

**Published:** 2025-07-03

**Authors:** Alejandra Perez, Elena Panagiotopoulou, Maria Christina Vourda, Mariana Pereira, Eamon McCrory, Ruth Roberts

**Affiliations:** 1 Postgraduate Studies, Anna Freud, 4–8 Rodney Street, London, N1 9JH United Kingdom; 2 Research Department of Clinical, Educational & Health Psychology, University College London, 1–19 Torrington Place, London, WC1E 7HB United Kingdom; 3 Department for Education, Sanctuary Buildings, Great Smith Street, London, SW1P 3BT United Kingdom

**Keywords:** Parenting confidence, Mother-infant relationship, Motherhood, Traumatic birth, Pregnancy, Prenatal expectations, Partner support, Prenatal representations, Qualitative, Longitudinal

## Abstract

**Background:**

Parents’ experiences and their relationship to their baby undergo various changes over the course of the first year. This is particularly the case for mothers, who still tend to take on the primary caregiver role in most families in the UK. Better understanding the changes mothers experience in the first year is of importance given the impact of the parent-infant relationship for children’s socio-emotional development.

**Methods:**

This qualitative longitudinal study explored first-time mothers’ experience of parenting confidence and relationship with their baby from their third trimester of pregnancy to the end of their babies’ first year of life. This study also examined trajectories of relevant consistent parenting factors: perceived social support, relationship with partner, expectations, and coping mechanisms. The sample consisted of ten first-time expectant mothers from a low-risk community urban sample, all White, the majority married or in committed relationships and with higher education. Participants were interviewed at four time periods (prenatal, 1-, 6-, and 12-months).

**Results:**

The findings indicate that in this homogenous, low-risk sample, most mothers’ parenting confidence improved with time, as did their relationship with their baby. However, most faced many changes in their experiences where, overall, the first six months after birth were the most challenging with many mothers feeling disconnected or having strong shifts in their views of relationship with their baby, feeling unsure about how to parent, having unmet prenatal expectations, and diminished partner support.

**Conclusion:**

This study demonstrates the complexity of change at multiple levels, both within individuals and within the 15 months of transitioning to motherhood. Consideration of these changes can help inform maternity services and mental health and social care professionals working with expectant parents and those in early parenthood to improve parenting confidence and mother-infant relationships.

**Supplementary Information:**

The online version contains supplementary material available at 10.1186/s12884-025-07683-0.

## Background

Although the significance of the parent-infant relationship for children’s socio-emotional development is well established [1], parents’ experiences and their relationship to their baby undergo various changes over the course of the first year. This is particularly the case for mothers, who still tend to take on the primary caregiver role in most families in the UK [2]. *Parenting confidence*, defined as one’s internal belief about their ability to be successful in their parenting role and foster their child’s development [3], has been found to influence various parent and child outcomes. The existing literature suggests that higher parental self-efficacy, often used interchangeably with parenting confidence, is associated with less depression, anxiety, and stress in parents [4, 5], more positive parenting practices [6, 7], as well as better child adjustment and overall development [5]. Maternal confidence has also been found to influence the mother-infant bond*,* with low maternal sense of competence intensifying bonding difficulties [8]. Supporting maternal bonding has also been found to enhance mothers’ self-confidence, suggesting that the two variables have a bidirectional relationship [9]. The early mother-infant bond, in other words a mother’s *relationship with the baby*, can have a long-term impact on the child’s social, emotional, and cognitive development [8, 10], as well as benefit the mother, for example, by protecting her from depression [11]. While these studies have indicated the importance of maternal confidence and the mother-infant relationship, these are not static factors, but ones that shift and change over time, especially as new expectant parents transition to parenthood in the early months [e.g. [12]].

Various factors impact maternal confidence and the mother-infant relationship. A large body of literature suggests that perceived social support influences parenting confidence [13–15], as well as mother-infant bonding [16–18]. This encompasses support from partner, family, friends, healthcare professionals and a wider social network, and it is multidimensional, ranging from practical support with pregnancy and childcare, to emotional and psychological support. Nevertheless, the source, content, and quality of this support play a crucial role [19, 20] and not all types of social support have positive outcomes. Interestingly, Perez and colleagues [15] found that the lack of childcare support during the most restrictive phase of the COVID-19 pandemic led to an increase in parenting confidence, suggesting that having too much unwanted advice and external support can make parents feel less confident in their ability to parent successfully. The quality of relationship and support received from partners is particularly important for mothers, influencing not only their confidence but also their relationship with the baby. Marital quality and emotional support from partners have been found to influence parental self-efficacy [21], whereas greater disappointment with the partner was generally associated with a weaker bond with the child [8]. Discrepancies between expectations toward the partner/child and reality were also found to intensify bonding difficulties with the baby [8]. Apart from unmet expectations, a lack of positive expectations about the relationship with the unborn baby in the prenatal period has been found to increase a mother’s risk of bonding difficulties [22, 23]. Furthermore, parenting efficacy has been found to predict positive coping strategies [24], although less is understood about how strategies to cope with stress may be related to mother-infant bonding [25].

The existing literature, therefore, suggests that maternal confidence and mother-infant relationship are interlinked and are important both for parental wellbeing, as well as children’s social, emotional, and cognitive development. Factors like perceived social support, relationship with partner, expectations, and coping styles seem to influence both mothers’ confidence and relationship with the baby. However, there is a lack of research exploring how these factors change and influence each other over time, and especially in first-time mothers, for whom these experiences are more novel and potentially more impactful. The aim of the current study was to examine changes in first-time mothers’ relationship with their baby and experience of parenting confidence from their third trimester of pregnancy to the end of their babies’ first year of life. This study also explored the influence of relevant consistent parenting factors’, such as perceived social support, relationship with partner, expectations, and coping mechanisms, with improved outcomes (i.e. how these may support or hinder maternal confidence and the mother-infant relationship).

## Method

### Setting, design and participants

This study forms part of a larger, mixed-methods Longitudinal Experiences and Adjustments in Parenthood Study (LEAPS) based at University College London (UCL) and Anna Freud (description of LEAPS in Appendix 1). Of the original 14 participants recruited during pregnancy, only 10 participants completed at least three of the four data points in the longitudinal study and were included in this study. Two the four participants who dropped out of the study did after the prenatal stage, and two after the 1-month follow-up. The reason for dropping out all participants gave was feeling overwhelmed with caring for their baby and not having time to do the interviews.

The current study employed a prospective, qualitative longitudinal design to capture changes as these were happening (Neale, 2021). Semi-structured, one-to-one interviews were conducted with a homogenous, low-risk sample of first-time mothers (*N* = 10) in London, UK at four different time points: third trimester of pregnancy, 1 month, 6 months and 12 months post-birth. Participants’ mean age was 33.9 years (*SD* = 3.88); all were White, and with one exception, married or in a committed relationship. Of the ten participants, eight had a higher education degree, one had an undergraduate degree, and one had 5 or more GCSEs or equivalent. Their depression and anxiety scores at the prenatal stage were minimal (BDI-II median = 9.50; SD = 2.94 and GAD-7 median 3; SD = 1.70). There was no data for the prenatal stage for one participant who couldn’t attend the interview and for another participant for the 12-month follow-up, who withdrew from the study at that point.

### Measures

The research team developed a semi-structured interview named the ‘Experiences of Parenthood Interview’ (EPI), which covers important areas identified in the literature, such as social support; relationship with partner; expectations of pregnancy; birth and baby; parental confidence; and what parents have found helpful and challenging at each stage of parenthood [e.g., 15]. EPI items were adjusted to each specific parenthood stage. For a full list of questions, details of interview development and LEAPS study see Appendix 1. Participants also completed an online socio-demographic survey, providing their age, ethnicity, and relationship and employment status. All procedures in this study were approved by UCL Research Ethics Committee (7683/003).

### Data analysis

The data from 38 interviews was analyzed to provide an in-depth look into parents’ lives, charting dynamic processes as they occurred [26]. Data was examined both at an individual level, longitudinally from pregnancy to when baby turned 1 year old, and across participants. The analysis used was Interpretative-Phenomenological Analysis (IPA)-informed thematic analysis, where we followed the principles of thematic analysis to identify and construct overarching patterns or themes across the dataset [27] and approached the analysis, interpretation, and conceptualization informed by IPA, an approach that surpasses the formation of generalizable statements and instead provides a fine-grained analysis of participants’ particular, individual experiences [28]. This qualitative method is based on the analysis of the participants’ narratives, is recommended for small sample sized interview-based studies of rich data, is attuned to social interaction and nuance, and incorporates participants’ thoughts and feelings as primary sources.

All analyses in this study were initially coded by two researchers, independently coding the same transcripts, then meeting to discuss the emergent themes identified, working in an iterative way, through inductive data engagement to refine themes and/or develop new themes, and work towards a shared understanding of the data. A third researcher then read all the themes developed by the first two coders to ensure accuracy of the description of themes and quotes to support the themes identified (see Appendix 2 for a completed consolidated criteria for reporting qualitative studies COREQ).

## Results

The results are presented in two sections: (i) qualitative thematic analysis of changes in parenting confidence and relationship with baby and (ii) qualitative thematic analysis of associated parenting factors and changes over time.

### Section I. Parenting confidence and relationship with baby over time

#### A. Trajectories of change in parenting confidence

Table 1 presents the three different trajectories of the changes in parenting confidence. All mothers described feeling confident by the end of their child’s first year. However, most of them (eight out of ten) felt unsure about how to parent in the first six months. Six of these eight mothers had started feeling unsure during pregnancy and their confidence developed gradually to feeling a strong confidence and this stemmed from the accumulated parenting experiences and seeing their babies developing well. In contrast, the two other mothers had felt very confident during pregnancy but lost all their confidence after a difficult birth and had to slowly build it up. A different trajectory identified was that of two mothers feeling moderately confident throughout the 15 months. Importantly, these mothers acknowledged the uncertainty of parenthood and the need for support throughout the year.

**Table 1:** Emergent themes for changes in parenting confidence

A. Changes in parenting confidence	*n* of participants contributing (*N* = 10)
*Improved greatly*	
A.1. Developing strong confidence by finding their own way with baby	6
*Maintained confidence*	
A.2. Stable confidence, relying on support and acknowledging uncertainty	2
*Decreased then improved*	
A.3. Loss of confidence at difficult birth and having to slowly build it up	2

#### A.1. Developing strong confidence by finding their own way with baby

Some parents had doubts about themselves as parents during pregnancy. For example, Clara felt “*generally confident*” but as the due date approached, she felt *“little wobbles… I do worry if I am cut to be a parent”.* Faye explained, *“what I don't feel confident about is the 24/7 relentlessness of being parent… You can't not be a parent at any point”.* Dara also felt *“not overly confident.”* At 1 month, mothers felt challenged by the unfamiliarity and fragility of their child. Clara explained, *“like the first time she was so little I was worried about dressing her. I felt like she was so dinky, and I didn't want to hurt her when I put some clothes on”.* However, meeting their babies’ needs and seeing them grow seemed to build experience and confidence. Dara described, “*but you just get on, and do what you need to do.”* By 12 months, Faye said, *“I feel like at this stage, we've kind of graduated to become professional parents… we've been through enough”.* Dara described, *“I think your confidence grows as you see her like developing, and getting stronger, and growing”.*

#### A.2. Stable confidence, relying on support and acknowledging uncertainty

Two mothers’ parenting confidence remained stable, albeit acknowledging uncertainty in the first year of parenthood. Since her pregnancy, Ana recognized the constant changes in parenthood, “*you just never know what’s gonna come at you”* but felt she could rely on support around her, *“a structured community around to lean on, ‘cause we, I can’t know everything”.* When baby arrived, Ana described her confidence as one that *“waxes and wanes”*. She relied on it to know how baby was doing, *“I’m doing great, she’s a happy baby, relaxed”,* yet acknowledging that the subjective experience of baby is to a certain degree unknown to her, *“we are seeing her from our perspective… and she’s having a different day every single day… what’s happening in the background? It’s a total mystery”.* By the end of the first year, Ana was feeling *“as confident as I did at the beginning… there are so many things you need to figure out and, you know, you’ve got to figure it out”.*

#### A.3. Loss of confidence at difficult birth and having to slowly build it up

Two mothers felt very confident when pregnant as they had previous experience with babies and children. For example, Bria explained, *“I’ve always been the one to mother [nieces and nephews] around me.”* However, both had unexpected complications during birth. The difficulties continued as both babies had to be kept in hospital for a few days, and they both struggled to settle them when crying. Both mothers described not feeling confident until their baby turned one year. Bria went from *“What do I do now?”,* when her baby was one month*,* to *“We know how that works now”* when baby turned one year*.* Eve also described changes in confidence over time, starting with *“I still don’t know that what I am doing is the right thing to do”* at one month, followed by *“I’m getting to know [baby] more and I feel like I’m getting to know myself more…”* at six months and finally, at one year, *“I feel a little bit more confident. And I feel like we get it right more than we get it wrong now… [baby] seems happier”.*

### B. Trajectories of change in relationship with baby

The different trajectories of change in the mothers’ relationship with baby are presented in Table 2. While most mothers (nine out of ten) reported an improved connection by the end of their baby’s first year, the thematic analysis revealed different trajectories. Three of these mothers described an oscillating sense of connection with their baby in the first year, starting with a mental representation of baby’s personality during pregnancy, to feeling disconnected at birth, to ending the first year with a strong connection to baby and fears of separation. Other mothers described an oddness when thinking about their baby during pregnancy and having a strong shift to feeling adoration of their baby after birth (at 1 month). Other mothers had a more gradual sense of getting to know and love their baby. Only one mother did not have an improved connection with her baby, describing struggles in the relationship throughout the first year.

**Table 2:** Emergent themes for changes in relationship with baby

B. Relationship with baby	*n* of participants contributing (*N* = 10)
*Improved*	
B.1. From imagination, to disconnection, to connection and fears of separation	3
B.2. From prenatal oddness to intense adoration	3
B.3. Gradually getting to know and love baby	3
*Did not improve*	
B.4. Ups and downs throughout	1

#### B.1. From imagination, to disconnection, to connection and fears of separation

Three mothers described an oscillating sense of connection with their baby in the first year. During pregnancy, they had a clear representation of their baby, which alluded to their imagined baby’s personality, *“I can definitely imagine him stealing the cats’ biscuits, pulling their tails”* [Bria] or “*we're a team [laughter], me and her”* [Faye]. However, both mothers struggled when feeling disconnected from their baby after birth. Bria expressed a feeling of “*not matching properly”* with baby and Faye described, “*I did feel slightly detached… just staring at her”.* By six months, both mothers reported feeling strong affection and enjoyment of their babies, especially around seeing them more mobile and interactive, *“she is very determined and she's constantly trying to reach for things that are out of her reach”* [Faye]. By 12 months, these mothers described preoccupations with limit-setting,* “as she learns things, that can be frustrating. She's learned how to open a certain sliding door, so you can no longer keep her out of an area just by shutting the door.”* [Bria]. With the growth and development of her baby, Faye struggled more with fears of separation, “*I really miss her. I just don't really like being apart from her, so I struggle with that”.*

#### B.2. From prenatal oddness to intense adoration

In contrast, three mothers described an oddness and sense of disbelief when thinking of their future babies when pregnant. Clara when seeing her baby on the scan, said, *“Oh my God what is that? Is that a peanut, did I swallow a big one? What's going on?”.* Dara conveyed the disconcerting oddness of feeling a body within her body, describing, *“even though I know what it is, it's strange that something is in there. Inside”.* Ivy described the experience *“me being like the host in Alien”* and the guilt of feeling this*, “instead of these magical bonding moments which maybe I should.”* These feelings shifted to intense love after birth. Clara, when breastfeeding, *“I swear I feel the hormones. I feel that rush of oxytocin and suddenly it's like an outburst of ‘Oh my God I love you so much’”.* For Dara, it took her a little longer but by six months she felt, “*completely devoted to her”.* All mothers described enjoying seeing their child develop, acquire new skills, and interacting more with them, “*everything is just a pleasure*” [Clara].

#### B.3. Gradually getting to know and love baby

For three mothers, getting to know their baby developed gradually from birth. During pregnancy scans, they felt relief of baby *“moving and healthy”* [Gail] and *“definitely a real live person inside there doing things.”* [Hope]. Ana also conveyed curiosity, recognizing so much still unknown about her baby, “*it just strikes what a little world she's got in there that you are like, ‘I am your world, but I have no idea what's going on’”.*

But it was after birth that they described a sense of getting to know and love their baby. Gail said, *“the love bit, like that grows. As you get to know them, that grows”.* Ana described the enjoyment of doing different activities together, appreciating times when it was just the two of them because *“my whole world can revolve around her”*. Hope felt pleasure when her baby responds to her, “*singing songs because he likes it and he’ll wiggle… it’s really, really nice*”.

By 12 months, these mothers continued to express their joy for moments of connection and interaction with them, as well as seeing their development, “*he's reading, he really participates now. He turns the pages…”* [Gail]. Ana said, “*we’ve built a nice kind of routine… but there’s always something new that she’s into or some new skill”.*

#### B.4. Ups and downs throughout

Eve described having “ups and downs” throughout the first year of her baby’s life. Her experience of seeing her baby on the scan was a mixture of surprise and relief, *“I wasn't really expecting it to look so baby-like*”. When born, Eve described feeling “*just like massive relief that she was safe and just pleasure that she was there really*”. However, she struggled with her baby’s lack of sleeping in the first year, “*I mean obviously there are ups and downs… I just enjoy every moment I can, and then the really tricky moments I just kind of try and work through them with her… I'm kind of pacing up and down and singing, ‘Please go to sleep’”.* By 12 months, feelings of reassurance of baby being healthy, as well struggling with baby’s ongoing sleeping problems continued, “…*not having such a tiny, tiny baby to worry about anymore, um, it's enjoyable… Um, so she's still not very keen on sleeping. Um, so getting her down to sleep for a nap or for bedtime, is still a bit of a battle*”.

### Section II. Associated parenting factors over time

The qualitative analysis of associated parenting factors (expectations; relationship with partner; socialization; and coping mechanisms) revealed different trajectories of change within the first year of baby’s life (see Table 3).

**Table 3:** Emergent themes and subthemes for associated parenting factors

Associated parenting factors	*n* of participants contributing (*N* = 10)
***1. Expectations***	
* Strong prenatal expectations*	
1.a. From disappointment of unmet prenatal expectations to more fulfilment	6
1.b. Expectations met but with unexpected emotional intensity	1
* Modest prenatal expectations*	
1.c. Modest expectations of just getting through each stage	3
***2. Relationship with partner***	*n* of participants contributing (*N* = 9)
* Improving*	
2.a. Developing a great partnership	2
* Declining*	
2.b. Diminished partner support	6
2.c. Baby receives all the attention and affection	1
***3. Socialization***	*n* of participants contributing (*N* = 10)
* Valuing*	
3.a. Valuing socialization throughout	7
* Ambivalent*	
3.b. Ambivalent about socialization	3
***4. Coping mechanisms***	*n* of participants contributing (*N* = 10)
* Anticipating & preparing*	
4.a. Anticipating difficulties and taking them in their stride	4
4.b. Anticipating and planning but struggling with ongoing challenges	1
4.c. Planning but letting go to be more in the moment	2
* Relying on others*	
4.d. From God, to others, to self	1
* Avoiding thinking*	
4.e. Avoiding thinking, minimizing, and “getting on with it”	1
4.f. Avoiding thinking and becoming overwhelmed with difficulties	1

#### Expectations

The results demonstrate a variety of responses in relation to expectations from the prenatal stage up to one year. Most mothers had many and strong expectations about pregnancy and birth and then became disappointed about their actual experiences during these periods. However, being exposed to baby and becoming a parent exceeded their expectations of parenthood. One mother, whose expectations were met, explained that the emotional intensity of parenthood was unexpected. Finally, some mothers had more modest expectations of just *“getting through”* each stage.

#### Relationship with partner

Most participants described growing difficulties in their couple relationship and their partner’s support being diminished over time in the first year. Some mothers developed a growing resentment and frustration about the imbalanced division of parenting, whilst others felt increasingly alone and distant from their partners. Only two participants described an improved relationship with their partner, yet in different ways, with one mother enjoying taking a lead in parenting, and the other developing a more balanced division of work with her partner and a strong sense of togetherness.

#### Socialization

Most participants valued socialization, with some describing it as an important aspect of sharing pregnancy news and others valuing meeting new parents who were going through similar experiences. Some expressed feelings of nostalgia for their pre-baby social life and support. Three participants expressed ambivalence about socialization which they found important but also challenging at times, for example, when wanting to spend alone time with their baby or feeling that some of the social support for parents can be *“doom and gloom.”*

#### Coping mechanisms

During pregnancy, participants described three main coping approaches: anticipating and preparing for the challenges ahead; relying on their faith and/or others to help them through difficulties; and avoiding thinking about problems (and this was due to a sense of having no control over the upcoming challenges). The first year of parenthood brought ongoing changes and challenges to all of them. Some relied on their initial coping mechanisms, while others adjusted these.

For a detailed description of each of the themes for associated parenting factors see Appendix 3).

## Discussion

Exploring changes in mothers’ parenting confidence and relationship with their babies over the first year of life indicated a complexity of change that is not picked up in studies looking only at specific time points. This study’s qualitative IPA-informed thematic analysis also illuminated the diversity of mothers’ individual experiences, approaches, and contextual factors that form part of each individual trajectory. Overall, in this low-risk sample, most mothers’ parenting confidence improved with time, as did their relationship with their baby. However, the majority faced many changes in their experiences where, overall, the first six months after birth were the most challenging with many mothers feeling disconnected or unknowing about their baby, unsure about how to parent, with unmet prenatal expectations and diminished partner support.

In terms of parenting confidence, for most mothers, this stemmed from their day-to-day parenting and resolving difficulties once the baby was born. It can be assumed that this built an individual knowledge and experience of their baby, which gave them confidence about parenting their own child. This finding is supported by a previous study [15] where the lack of external advice and support during the lockdown of Covid-19 presented challenges for new parents but it also helped them feel more confident by having to engage with their baby on their own. In the present study, when mothers began seeing their baby happy and developing, they described feeling more confident about future challenges and uncertainties, as well as the changing needs of their baby. This trajectory contrasted with the two mothers who had very difficult births and lost the strong parenting confidence they felt during pregnancy. A previous qualitative study found that mothers who experienced birth trauma felt a lack of parenting confidence and disconnection to their babies [29]. While there is no consistent definition of birth trauma, it has been broadly defined in research as a birth that is experienced by mothers as distressing because of a perception of ‘actual or threatened injury or death to the mother or her baby’ [30]. An additional aspect which might indicate the complexity of emotional recovery for mothers who experience a traumatic or difficult birth is having had previous expectations of parenthood and having felt confident during pregnancy. Molloy and colleagues [29] also reported that mothers struggled due to feeling unable to fulfil their own expectations of being a mother. Support for mothers who have had traumatic births should consider these two different (albeit intermixed) aspects: the recovery from the frightening experience of birth and the grief and mourning of their previous expectations of birth and themselves as parents to help restore their parenting confidence.

Another important finding of this study was the changes in mothers’ experiences of their relationship with the baby, which for some, began from pregnancy by imagining their future baby. Prenatal representations of the unborn baby or fetus have been considered important as studies have found these to be remain stable straight after birth [31], at four to six months after birth [32, 33], and even predictive of future mother-infant relationships at 12 months [34]. These findings contrast with the present study where mothers described many changes in their feelings towards baby in the first year. Interestingly, mothers who during pregnancy had the clearest mental representation of their baby (which included baby’s personality and positive feelings towards baby), had a strong shift to feeling disconnected at birth, and ended the first year with a strong connection to baby but with fears of separation (the trajectory theme ‘From imagination, to disconnection, to connection and fears of separation’). In contrast, mothers who had a more unclear representation of their unborn baby during pregnancy yet curious and with positive feelings of baby had a more gradual shift to getting to know and love their baby. Another different trajectory was of expectant mothers who described a sense of *“oddness”* and *“strangeness”* when thinking about their future baby but shifted quickly to intense adoration after birth. Given the present study’s in-depth interviews and collection of data at four different time points, it was possible to capture these changes in more detail. It is important to highlight the positive aspects found in expectant mothers’ more unclear but positive representations of their unborn baby which is not described in previous research. Further research can help understand whether a more unknowing but curious stance towards the fetus can help mothers once the baby is born to be more open, flexible, and adapt to their babies’ individual needs and challenges brought by parenthood. Future research should consider not only whether expectant parents have mental representations of the fetus or not and whether these are positive or negative, but also to explore qualitatively and longitudinally changes in the characteristics of representations of their babies, the intensity of the feelings, whether they include the baby’s personality and whether representations are held rigidly which could make the reality of the baby once born more difficult to adapt to. Further follow-ups of the present study can explore whether mothers who shifted from feelings of *“oddness”* to intense adoration soon after birth could be a defense or coping mechanism against the disconcerting prenatal feelings.

Regarding associated parenting factors, more than half of the participants in this study felt disappointed of unmet prenatal expectations of birth, the baby, and/or parenthood that made the postpartum and first year more challenging. Even mothers whose expectations were met described feeling surprised at the intensity of their emotions, indicating how preparation for parenthood is often done at a cognitive level but not at an emotional one. Despite the small cohort, it is worth noting that most mothers with partners felt a decline in partner support in the first year. This is an important finding as research has shown how social support, including that from partner, is a strong factor in parents’ confidence [13–15], and bonding with their baby [16–18]. Biehle and Mickelson [35] compared prenatal expectations and postnatal experiences of division of childcare in parents and found that mothers felt fathers did less than they expected, and fathers reported over-met expectations with mothers doing more than they had anticipated. Importantly, these unmet expectations were a strong prediction of depression and relationship satisfaction, in opposite directions for mothers and fathers. The parents in the present study were not going through separation or divorce, nor had any other significant negative life events challenging the couple. The diminished support from partner was in relation to childcare and some mothers expressed feeling alone with this responsibility. Special attention should be given to expectant parents, where discussions on their expectations of childcare and planning of division of tasks should be encouraged by health professionals during pregnancy and support should be made available in the first six months to a year. Additionally, most mothers valued and missed socializing in the first year. Their appreciation of social relations ranged from being an important aspect of sharing pregnancy news to the importance of meeting new parents who were going through similar experiences, demonstrating further the need for support during this time.

As to coping mechanisms used by mothers in this study, these were variable and changed in the first year. The first year of parenthood brought ongoing changes and challenges to all parents interviewed, from sleep deprivation and struggles with breastfeeding, to difficult births and medical complications in the first months. Some relied on their initial coping measures and approaches while others adjusted these. Only two mothers avoided thinking about difficulties (by denying, forgetting, or minimizing) as a coping strategy. By 6 months, one of these mothers began to acknowledge difficulties more and felt able to manage these, as she put it, *“just get on with it”.* The other of these two participants began to feel overwhelmed by 12 months, and she felt she had no sense of control or way to face these difficulties. However, most mothers anticipated and prepared for difficulties. One mother who planned for difficulties struggled with the constant changes her growing baby entailed, and this meant she couldn’t plan as she did before. Participants who found it easier to deal with the changes and challenges of parenthood were those that did not plan too rigidly for upcoming problems and took them more *“in their stride”* and felt able to listen to themselves and their baby. These findings indicate that parents who can recognize the ever-changing nature of parenthood and the individuality of their own baby find it easier in the first year. Similarly, a study by Borelli and colleagues [36] looked at expectant mothers’ coping strategies for childbirth and found they described the importance of being flexible regarding the birth plan and changes needed as birth entailed much uncertainty.

### Strengths and limitations

This is the first known study of its kind, investigating closely and longitudinally the changes in mothers’ parenting confidence, relationship with their baby, and associated factors in the first year. The strengths of this study are its in-depth, individual interviews collected prospectively at four different time points as mothers transitioned to early parenthood, capturing changes and dynamic processes as they were happening, and the detailed qualitative analysis at an individual level, case by case, longitudinally, and across participants. However, given that four participants withdrew from the study given how overwhelmed they felt after the birth of their baby and the time required to do the interviews, this type of study will not be able to collect information from parents with these characteristics. While small sample size and homogeneity are recommended for IPA-informed thematic analysis to ensure in-depth analysis [27, 28], this limits the generalizability of our findings. Future studies should aim to focus on different socio-demographic samples to build a larger, more diverse understanding of parents’ experiences.

## Conclusion

In conclusion, this study shows the complexity of change at multiple levels, both within individuals and within the 15 months of transitioning to motherhood. While in this low-risk sample most mothers’ parenting confidence and relationship with their baby improved by the end of their baby’s first year, the first six months were the most challenging. We hope findings can help inform maternity services and mental health and social care professionals working with expectant parents and those in early parenthood. How preparation for parenthood should include discussions of their expectations of childcare division and the importance of flexibility in their expectations of parenthood and their baby as it allows greater adaptability to the changing and individual needs of their baby. Support for women who had traumatic births should include not only help with the traumatic experience but also help those who are suffering the loss of previous expectations of themselves, birth, and/or their baby to be able to regain confidence and bond with their baby. Partner and social support for new mothers is important as is the exposure to baby and doing the day-to-day parenting tasks to build parenting confidence and relationship with their baby. Future research should explore in more depth the quality and changes in parents’ prenatal representations of their baby and whether this has an impact on their future relationship with them.

## Supplementary Information

Supplementary Material 1.

Supplementary Material 2.

Supplementary Material 3.

## Data Availability

The data that support the findings of this study are available from the corresponding author, AP, upon reasonable request. Following guidance from UCL’s Ethics Committee, we included the following in the Participant Information Sheet: only UCL researchers will have access to the anonymous data which will be deleted after 10 years. If access to the raw data is required, approval from the UCL Ethics Committee will be requested.
