# Supplementary material for: Trajectories of change in mothers’ parenting confidence and relationship with baby: a 15-month qualitative longitudinal study
Source: BMC Pregnancy Childbirth. 2025 Jul 3;25:709. doi: 10.1186/s12884-025-07683-0 (PMC12224638; doi:10.1186/s12884-025-07683-0)
Supplement: Supplementary file 1 — Supplementary Material 1. [file 12884_2025_7683_MOESM1_ESM.docx]

**Appendix 1: Lab of Experiences and Adjustments in Parenthood (LEAP) and Interview Protocols**

**Lab of Experiences and Adjustment in Parenthood (LEAP)**

The Lab of Experiences and Adjustments in Parenthood (LEAP) is a research lab that aims to better understand parents’ subjective experiences through different stages of parenthood. Our work also investigates the complex interconnections of parent, infant, and social/contextual factors in the parent-child relationship and children’s mental health, using a range of qualitative and quantitative methods.

LEAP is based at University College London and Anna Freud and was established in early 2020 by Dr Alejandra Perez (Principal investigator), Dr Ruth Roberts (Researcher) and Dr Elena Panagiotopoulou (Researcher), later welcoming Maria-Christina Vourda (Research Assistant) and Mariana Pereira (Researcher) as core members.

LEAP includes a primary longitudinal research project, along with several sub-projects and satellite projects. All research projects have received ethical clearance from UCL (Ethics ID number: 7683/003). Below is a summary of all LEAP projects.

*Longitudinal Experiences and Adjustments in Parenthood Study (LEAPS)*

LEAPS is a longitudinal mixed-methods study that investigates expectant and new parents’ experiences of parenthood, as well as individual and contextual factors playing a role in the parent-child relationship and child’s development. The study recruits expectant parents in their 3rd trimester of pregnancy (prenatal stage) with follow-ups at 1-, 6-, 12-, 18-months, 2-, 3-, 4-, and 5-years. LEAPS employs a mixed-methods investigation, including semi-structured interviews, self-report surveys, and observational measures.

*LEAPS sub-projects*

To better understand the experiences of a more diverse population and hard-to-reach groups, our lab has developed six sub-projects within LEAPS, using the same protocol:

- - Pregnancy and parenthood in non-traditional families, recruiting transgender and non-binary expectant parents.
  - Pregnancy and parenthood in expectant fathers, this project was developed in response to the very low engagement of fathers in our pilot study (one father only, who later withdrew). We hope that field notes will provide an insight on potential barriers and facilitators to fathers’ participation in research.
  - Pregnancy and parenthood in low-income families
  - Pregnancy and parenthood in Black families
  - Pregnancy and parenthood when supported by surrogacy

*LEAP satellite projects*

- COVID-19 and Parenthood Project

The aim of this research project is to investigate the impact of COVID-19 and associated restrictions on expectant and new parents. Two mixed-methods studies were carried out: The first collected data during the first and most restrictive phase of the nationwide lockdown in the UK (April-May 2020) and produced one publication. The second study collected data one year later (April-October 2021) and looked at the impact of prolonged restrictions on expectant and new parents’ experience of control over decisions regarding pregnancy and parenting.

- Parenthood and the Bodily Self Project

The aim of this project is to investigate the impact of the transition to parenthood on new mothers’ bodily self (i.e., body image, bodily awareness, and body ownership).

- Protective and Risk Factors in Early Patenting Project

This project focuses on protective and risk factors of positive parenting in early infancy. As part of this project, we conducted a systematic review of parent, child and contextual factors related to positive parenting in the first year of an infant's life.

- Maternal and infant temperament:

This project aims to 1) uncover the neural markers of temperament traits and behaviors in infant and adults, 2) identify behavioral and neural correlates between mothers and infants, and 3) explore how maternal temperament impacts the subjective perception of infant temperament. To uncover this myriad of questions, a mixed-methods design will be used, integrating self-report questionnaires, observational measures, qualitative interviews, and electrophysiological data.

**Interview Protocols**

*PRENATAL stage interview*

| **INTRODUCTION**   - Explain purpose of the interview - Address terms of confidentiality - Remind free to stop or withdraw from study at any point - Ask if any questions before starting |
| --- |
| **1. Can you tell me what it was like when you found out you were pregnant/expecting (if partner)?**  *Follow-up questions*:   - Do you remember what was your first reaction? - AND (if not answered) What did you think/felt then? |
| **2. What was the first trimester of pregnancy like for you?**  *Follow-up questions*:   - What kinds of things made this period easier for you? - What kinds of things made this period more challenging for you? - Was this experience what you had expected/imagined it would be?   To note:  Are any significant others mentioned as helpful/supportive? |
| **3. What was the second trimester of pregnancy like for you?**  *Follow-up questions:*   - What kinds of things made this period easier for you? - What kinds of things made this period more challenging for you? - Was this experience what you had expected/imagined it would be? |
| **4. Can you tell me about your experience now (third trimester)?**  *Follow-up questions:*   - What kinds of things made this period easier for you? - What kinds of things made this period more challenging for you? - Was this experience what you had expected/imagined it would be? - Could you talk me through your day yesterday so I can get an idea of your life at the moment? |
| **5. Do you remember the first time you felt your baby?**  *Follow-up questions*:   - What was it like? - How did it feel? - What were the thoughts that went through your mind? |
| **6. Do you remember the first time you heard your baby’s heartbeat?**   - What was it like? - What did it sound like? - What were the thoughts that went through your mind? |
| **7. Do you remember the first time you saw your baby in a scan?**   - What was it like? - What did your baby look like? - What were the thoughts that went through your mind? |
| **8. What kinds of things are the most challenging for you at the moment?** |
| **9. Can you tell me about any fears you have about the next stage in your life?** |
| **10. Can you tell me about any hopes you have about the near future?** |
| **11. What kinds of things do you hope for for your future baby?** |
| **12. How do you imagine your baby will be like when he or she is 6 months old?**  *Probe:*   - How do you imagine he or she will be with you, with others, playing? |
| **13. How confident do you feel about being a parent?**   - What sorts of things do you think you will find easy? - What sorts of things do you think you will find rewarding? - What sorts of things do you think you will find challenging? |
| **14. Since becoming pregnant/expecting your baby, have your thoughts or feelings about parenting changed – and if so, how?** |
| **15. Could you tell us about your motivation for participating in this study?** |
| **END**  **Well, that is the end of this interview. Is there anything else that you’d like to say about your experience that I might have missed?**  **Thank you.** |

*1-month follow-up interview*

| **INTRODUCTION** | |
| --- | --- |
| **1. Can you tell me what the birth was like?**  (If not answered) Follow-up question(s):   - Do you remember what thoughts and feelings you had during labour? - And during birth? - (If not answered) Did you feel frightened at any point during labour or birth? - What kinds of things made this experience easier for you? - What kinds of things made this experience more challenging for you? - Was the birth what you expected? |  |
| **2. What was your experience of the health professionals surrounding the birth?** |  |
| (If relevant)  **3. What was your experience of your partner during birth?** |  |
| **4. Can you tell me your thoughts and feelings when you first saw your baby?**  Follow-up questions:   - Was this experience what you expected? |  |
| **5. And what thoughts and feelings did you have when you first held your baby?**  Follow-up questions:   - Was this experience what you expected? |  |
| **6. How are the first few weeks of parenthood been like for you?**  Follow-up question(s):   - What kinds of things made this period easier for you? - What kinds of things made this period more challenging for you? - Was this experience what you had expected/imagined it would be?   *To note:*  *Are any significant others mentioned as helpful/supportive?* |  |
| **7. How do you feel when you are with baby?**  *(If not answered) Follow-up question*:   - And how do you feel when you are with baby on your own? |  |
| **8. How has feeding been like?**  *To note:*  *Holding and feelings of connection during feeding* |  |
| **9. How has baby sleeping been like?** |  |
| **10. How has sleeping been like for you?** |  |
| **11. What is your relationship with your partner like now (now with your baby)? - [IF relevant]**  *Follow-up questions:*   - What things have changed? - What things have stayed the same? |  |
| **12. How confident do you feel in your role as a parent?** |  |
| **13. If you could ask for anything to help you/support you during this period, what would it be?** |  |
| **14. Since becoming a parent, have your thoughts or feelings about parenthood changed – and if so, how?** |  |
| **END**  **Well, that is the end of this interview. Is there anything else that you’d like to say about your experience that I might have missed?**  **Thank you.** |  |

*6 months follow-up interview*

| **INTRODUCTION** |
| --- |
| **1. Can you tell me about your baby:**   - **What sorts of things does he/she enjoy?** - **What sorts of things does he/she dislike?** |
| **2. What types of things do you enjoy doing with your baby?** |
| **3. What types of things do you find challenging/a struggle doing with your baby?** |
| (If relevant)  **4. Do you have any concerns about your baby?**  (If not answered) Follow-up question(s):   - Do you have any concerns about his/her development? - Do you have any concerns about his/her health? |
| **5. How has baby’s feeding been like?** |
| **6. How has baby’s sleeping been like?** |
| **7. Can you tell me what the last 5 months have been like for you as a parent?**  (If not answered) Follow-up question(s):   - What kinds of things made this experience easier for you? - What kinds of things made this experience more challenging for you? - Has this [experience] been what you expected? |
| **8. How have you been feeling generally?**   - **Have you been feeling yourself?** |
| **9. What is your relationship with your partner like now (now with your baby)?**  *Follow-up questions:*   - What things have changed? - What things have stayed the same? - Is this what you expected? |
| **10. Do you take turns or divide the caring tasks of your baby with your partner?**  *(If not answered) Follow-up question*:   - What types of things does your partner help you with? - Is there anything you’d like your partner to help you with? |
| **11. Do your parents, in-laws, relatives, or friends help you with caring for your baby?**  *(If not answered) Follow-up question*:   - What types of things do your parents (etc.) help you with? - Is there anything you’d like your parents (etc.) to help you with? |
| **12. How do you imagine your family will be like in 10 years?**  *(If not answered) Follow-up question*:   - How do you imagine your baby will be like in 10 years? - How do you imagine your relationship with your partner will be like in 10 years? |
| **13. How confident do you feel as a parent?** |
| **14. If you could ask for anything to help you/support you during this period, what would it be?** |
| **15. Since becoming a parent, have your thoughts or feelings about parenthood changed – and if so, how?** |
| **END**  **Well, that is the end of this interview. Is there anything else that you’d like to say about your experience that I might have missed?**  **Thank you.** |

*12 months follow-up interview*

| **INTRODUCTION** |
| --- |
| **1. Can you tell me what types of things do you enjoy doing with your baby?** |
| **2. What types of things do you find challenging/a struggle doing with your baby?** |
| **3. How has baby’s moving around or crawling been like?**   - Has baby began to walk/first steps? - Probe: Do you have any concerns about this? |
| **4. How has baby’s sleeping been like?**   - Probe: Do you have any concerns about this? |
| **5. How has your baby’s eating been like?**   - Probe: Do you have any concerns about this? |
| **6. How do you find your baby’s communication with you?**   - Has your baby said their first word? If yes, what was it? - Do you feel you understand what your baby is wanting to communicate? - Do you feel that your baby understands you? - Probe: Do you have any concerns about this? |
| **7. Have you found the need to set boundaries or limits with your baby?**   - What has this been like for you? - How has your baby responded to this? - How do you feel your baby experiences this? |
| **8. Can you tell me what the last few months have been like for you as a parent?**  (If not answered) Follow-up question(s):   - What kinds of things made this experience easier for you? - What kinds of things made this experience more challenging for you? - Has this [experience] been what you expected? |
| **9. What is your relationship with your partner like now (now that you’ve had a baby)?**  *Follow-up questions:*   - What things have changed? - What things have stayed the same? - Is this what you expected? |
| **10. What is it like when the three of you are together?**  *Follow-up questions:*   - What things have changed? - What things have stayed the same? - Is this what you expected? |
| **11. Can you tell me of times when you are with your partner (dad) and your baby needs your attention? Does this change how you are with your partner (dad) in this moment and, if so, how?**  *Prompt:*   - Can you give me an example?   *(If not answered) Follow-up question(s):*   - How do you feel during these moments? |
| **12. Can you tell me of times when you are with the baby alone and your partner (dad) comes in? Does this change how you are with the baby in this moment and, if so, how?**  *Prompt:*   - Can you give me an example?   *(If not answered) Follow-up question(s):*   - How do you feel during these moments? - What do you think your partner (dad) might be feeling/thinking when he does this? |
| **13. Can you tell me of times when your partner (dad) is with the baby on their own and you come in? Do you feel that this changes how your partner (dad) is with your baby in this moment and, if so, how?**  *Prompt:*   - Can you give me an example?   *(If not answered) Follow-up question:*   - How do you feel during these moments? |
| **14. Do you take turns or divide the caring tasks of your baby with your partner?**  *(If not answered) Follow-up question*:   - What types of things does your partner help you with? - Is there anything you’d like your partner to help you with? |
| **15. Do your parents, in-laws, relatives, or friends help you with caring for your baby?** |
| **16. How do you imagine your family will be like in 10 years?**  *(If not answered) Follow-up question*:   - How do you imagine your baby will be like in 10 years? - How do you imagine your relationship with your partner will be like in 10 years? |
| **SIBLING SUPPLEMENT (can move to 6 months):**  **17. Have you thought about having another baby? OR (if pregnant) How have you been feeling about the impending arrival of your second baby?**   - How has partner been feeling about this? - (if pregnant) Has baby no. 1 been told? Do you think baby no 1. is aware of this second baby? |
| **18. Have you thought about going back to work?**   - Have you made plans on when to return? (note time/when and childcare arrangements) - How do you feel about this? - (if they have already gone to work) - How has it been/felt being separated from your baby? |
| **19. How confident do you feel now as a parent?** |
| **20. If you could ask for anything to help you/support you during this period, what would it be?** |
| **21. Now that your baby is 1 year old, have your thoughts or feelings about parenthood changed – and if so, how?** |
| **END**  **Well, that is the end of this interview. Is there anything else that you’d like to say about your experience that I might have missed?**  **Thank you.** |
