# Supplementary material for: Trajectories of change in mothers’ parenting confidence and relationship with baby: a 15-month qualitative longitudinal study
Source: BMC Pregnancy Childbirth. 2025 Jul 3;25:709. doi: 10.1186/s12884-025-07683-0 (PMC12224638; doi:10.1186/s12884-025-07683-0)
Supplement: Supplementary file 2 — Supplementary Material 2. [file 12884_2025_7683_MOESM2_ESM.docx]

**Appendix 2: Consolidated criteria for reporting qualitative studies (COREQ): 32-item checklist**

Developed from:

Tong A, Sainsbury P, Craig J. Consolidated criteria for reporting qualitative research (COREQ): a 32-item checklist for interviews and focus groups. *International Journal for Quality in Health Care*. 2007. Volume 19, Number 6: pp. 349 – 357

| **No. Item** | **Guide questions/description** | **Notes** |
| --- | --- | --- |
| **Domain 1: Research team and reﬂexivity** | | |
| *Personal Characteristics* | | |
| 1. Interviewer/facilitator | Which author/s conducted the interview or focus group? | The interviews were conducted by most of the authors (AP, EP, MC, MP, and RR) and students of the MSc in Early Child Development and Clinical Practice (University College London and Anna Freud). |
| 2. Credentials | What were the researcher’s credentials? e.g. PhD, MD | At the time of the study, authors AP, EP, RR, and EMC all had PhDs. MP submitted her PhD within 6 months and MCV had an MSc qualification, and in the process of applying for a PhD at the time of the study. |
| 3. Occupation | What was their occupation at the time of the study? | AP was Programme Director of the MSc in Early Child Development and Clinical Practice, Head of the research lab LEAP (Lab of Experiences and Adjustments in Parenthood, based at Anna Freud), Parent-Infant Psychotherapist at Anna Freud, and Psychoanalyst in private practice. EP was Deputy Director of the MSc in Early Child Development and Clinical Practice, and in training for Couple & Individual Psychodynamic Psychotherapist. RR was Senior Research Tutor for the MSc in Early Child Development and Clinical Practice. MCV was research assistant for the research lab LEAP. MP was Course and Research Tutor and Module Lead for the MSc in Early Child Development and Clinical Applications. EMC was Head of Postgraduate Studies and Clinical Psychologist at the time of the study. |
| 4. Gender | Was the researcher male or female? | Except for author EMC, all researchers and interviewers were female. |
| 5. Experience and training | What experience or training did the researcher have? | All researchers had qualitative methods and interviewing training prior to collecting data. AP, EP, RR, and EMC all had previous experience of qualitative methodology. |
| *Relationship with participants* | | |
| 6. Relationship established | Was a relationship established prior to study commencement? | No prior relationship existed between the researchers and participants. The research team did not have any contact with participants prior to obtaining informed consent. |
| 7. Participant knowledge of the interviewer | What did the participants know about the researcher? e.g. personal goals, reasons for doing the research | The participants were given the name of the researcher prior to the interview. The information sheet and flyer included information about the study’s research team and aim of the study. Once participants agreed to participate in the study, an interview date was arranged by email and participants were told the name of their interviewer. No other characteristics were stated about the interviewer. |
| 8. Interviewer characteristics | What characteristics were reported about the interviewer/facilitator? e.g. Bias, assumptions, reasons and interests in the research topic | All interviewers that formed part of the research study had interest in the topic. |
| **Domain 2: study design** | | |
| *Theoretical framework* | | |
| 9. Methodological orientation and Theory | What methodological orientation was stated to underpin the study? e.g. grounded theory, discourse analysis, ethnography, phenomenology, content analysis | The methodological orientation was phenomenological. We conducted an Interpretative Phenomenological Analysis-informed reflexive thematic analysis and analysed the data in an inductive manner. |
| *Participant selection* | | |
| 10. Sampling | How were participants selected? e.g. purposive, convenience, consecutive, snowball | Participant selection was purposive. |
| 11. Method of approach | How were participants approached? e.g. face-to-face, telephone, mail, email | There were several ways participants were approached. One was through direct contact with relevant groups to invite them to the study and hand-out flyers and information sheets. We had identified several relevant groups in London, UK (i.e. Camden baby feeding team, a locum sonographer, Linden children’s Centre antenatal group, NCT). The second way was through indirect contact, where leaders of such groups, after speaking with us researchers and agreeing to help, would hand out potential participants our flyers and information sheets. If we were given permission, we also uploaded our flyers to their online (social media) groups. The third way is through social media posts (our research lab LEAP has a study Instagram, X, and Facebook accounts that were disseminated broadly). |
| 12. Sample size | How many participants were in the study? | 10 participants. |
| 13. Non-participation | How many people refused to participate or dropped out? Reasons? | 4 participants dropped out. Of the original 14 participants recruited during pregnancy, only 10 participants continued in the study for the 12-month follow-up and were included in this study. Two the four participants who dropped out of the study did after the prenatal stage, and two after the 1-month follow-up. The reason for dropping out all participants gave was feeling overwhelmed with caring for their baby and not having time to do the interviews. |
| *Setting* | | |
| 14. Setting of data collection | Where was the data collected? e.g. home, clinic, workplace | Interviews were conducted online. |
| 15. Presence of non-participants | Was anyone else present besides the participants and researchers? | No. |
| 16. Description of sample | What are the important characteristics of the sample? e.g. demographic data, date | Participants’ mean age was 33.9 years (*SD* = 3.88); all were White, and with one exception, married or in a committed relationship. Of the ten participants, eight had a higher education degree, one had an undergraduate degree, and one had 5 or more GCSEs or equivalent. Their depression and anxiety scores at the prenatal stage were minimal (BDI-II median = 9.50; SD = 2.94 and GAD-7 median 3; SD = 1.70). |
| *Data collection* | | |
| 17. Interview guide | Were questions, prompts, guides provided by the authors? Was it pilot tested? | Our research team developed a semi-structured interview protocol named the ‘Experiences of Parenthood Interview’ (EPI), which covers important areas identified in the literature, such as social support; relationship with partner; expectations of pregnancy; birth and baby; parental confidence; and what parents have found helpful and challenging at each stage of parenthood. EPI questions and prompts were adjusted to each specific parenthood stage. We have included a full list of questions of each interview protocol, details of interview development, and LEAPS study in Appendix 1.  The EPI prenatal protocol was pilot tested with two new mothers, friends of the researchers but outside the field of psychology to ensure the language was accessible, the questions clear, and that the interview had a coherent flow. |
| 18. Repeat views | Were repeat interviews carried out? If yes, how many? | No. |
| 19. Audio/visual recording | Did the research use audio or visual recording to collect the data? | Interviews were conducted online and audio and video recorded via a secure Teams channel. Interviews were then transcribed verbatim. |
| 20. Field notes | Were ﬁeld notes made during and/or after the interview or focus group? | No. |
| 21. Duration | What was the duration of the interviews or focus group? | The duration of the interviews ranged from 30 min to 1 hr. |
| 22. Data saturation | Was data saturation discussed? | Data saturation was discussed during data collection and data analysis, to determine the depth, clarity, and nuance of the themes, not to determine an end point of no new themes. Data saturation to determine an end point is a contested concept within IPA and reflexive thematic analysis (see Braun & Clarke, 2021) in the sense that the themes try to capture participants’ subjective experiences across time and require a level of reflexivity and interpretation from the researcher, a fixed end point of no new themes is not considered possible (Low, 2019). |
| 23. Transcripts returned | Were transcripts returned to participants for comment and/or correction? | No. |
| **Domain 3: analysis and ﬁndings** | | |
| *Data analysis* | | |
| 24. Number of data coders | How many data coders coded the data? | There were two coders (AP and EP) of the data and a third researcher (MCV) reviewed the coding and analyses to ensure accuracy. |
| 25. Description of the coding tree | Did authors provide a description of the coding tree? | We provide tables of our main themes and subthemes (coding tree) in the results section, which indicate the hierarchical relationship of the emergent themes and subthemes identified. |
| 26. Derivation of themes | Were themes identiﬁed in advance or derived from the data? | The themes were derived from the data in an inductive manner. |
| 27. Software | What software, if applicable, was used to manage the data? | The data analysis was carried out manually by the researchers, there was no software used to manage the data. |
| 28. Participant checking | Did participants provide feedback on the ﬁndings? | No. |
| *Reporting* | | |
| 29. Quotations presented | Were participant quotations presented to illustrate the themes/ﬁndings? Was each quotation identiﬁed? e.g. participant number | Key findings are supported with illustrative quotes from the participants (these are referenced in the manuscript, in quotes and in italics). We also provide additional supporting quotes in Appendix 3. |
| 30. Data and ﬁndings consistent | Was there consistency between the data presented and the ﬁndings? | All the findings of this study were derived from the data and all themes are supported by quotes. |
| 31. Clarity of major themes | Were major themes clearly presented in the ﬁndings? | Yes, emergent themes are presented in the results section, supported by illustrative quotes. |
| 32. Clarity of minor themes | Is there a description of diverse cases or discussion of minor themes? | Yes, we include description of all participants. Emergent themes and subthemes were developed irrespective of whether they represented the majority or only one or two of the participants, and the number of participants each theme refers to is indicated in the results section. |

**References**

Braun, V. & Clarke, V. (2021). To saturate or not to saturate? Questioning data saturation as a useful concept for thematic analysis and sample size rationales. *Qualitative Research in Sport, Exercise and Health*, 13:2, 201-216.

Low, J. (2019). A pragmatic definition of the concept of theoretical saturation. *Sociological Focus*, 52 (2): 131–139.
