# Supplementary material for: Trajectories of change in mothers’ parenting confidence and relationship with baby: a 15-month qualitative longitudinal study
Source: BMC Pregnancy Childbirth. 2025 Jul 3;25:709. doi: 10.1186/s12884-025-07683-0 (PMC12224638; doi:10.1186/s12884-025-07683-0)
Supplement: Supplementary file 3 — Supplementary Material 3. [file 12884_2025_7683_MOESM3_ESM.docx]

**Appendix 3: Thematic analysis of associated parenting factors**

***Expectations***

**1.a. From disappointment of unmet prenatal expectations to more fulfilment**

Six participants described a transition from disappointment about unmet expectations of pregnancy and/or birth to a more fulfilling experience of parenthood. Three participants described their disappointment about their pregnancy. Faye expected it to be more social and *“that my friends and family would be around a lot more helping do stuff (...) But the third trimester has been a lot more a lot more private than I thought it would be”.* Hope and Dara found their pregnancy more difficult than expected. For Hope *“when baby reaches up and you kind of get a foot in in your rib cage”* it was more uncomfortable than she expected it and it *“started a little bit sooner than perhaps I expected them* *to come”.* All six participants described failed expectations of birth. Dara, who had an unplanned caesarean section, was *“kind of panicked because I was like ‘I don't know about this’”* and felt like *“nothing was how I expected from the whole experience”,* like Gail, who also felt, *“like it had gone from being completely ideal to completely opposite”.* Joy explained that she always thought it would be painful but she *“didn't expect it to be so painful”.*

Despite the initial disappointment, for all six participants parenthood exceeded their expectations, describing it as a fulfilling experience. At one month, Faye said:

*I expected [parenthood] to be really difficult. Uhm, I expected me and my partner to argue a lot. I expected to feel quite alone and unsupported. I expected to feel depressed. I expected to feel exhausted beyond anything I'd experienced before. None of that happened.*

At six months, Gail said “*I honestly didn't think that it would be this good. And people told me like it would be really hard (...) and I just don't think it's been like that at all (...) It's just been so much better than I expected.”* Others thought it was “*both harder and more rewarding than I thought it would be” (Bria, six months)*. Hope, at 12 months, had a similar take on parenthood: *“it's more amazing than I thought it was going to be (…) But then, it's also harder than I thought it was going to be”.*

**1.b. Expectations met but unexpected emotional intensity**

One participant described the experience of having all her expectations met but being surprised by the intensity of her emotions. Eve explained that her pregnancy was *“pretty much what I expected”*. At one month, she said, *“some things are what I expected, but like times a million”* and, at six months, the feeling persisted: “*Everything that's happened as a parent has been something that I've expected, but I just hadn't expected the intensity to which they're like”.* This did not change at 12 months: *“It has been what I expected”* and adding *“I have been surprised by the enormity of it (...) how strong my feelings would be. And I didn't realize how, um, kind of overpowering the emotional experience would be”.*

**1.c. Modest expectations of just getting through each stage**

Three participants had no expectations and just wanted to get through each stage. Ivy explained that she had no expectations for her pregnancy: *“I didn't really have any expectation. To get through it really is my expectation”.* Clara explained that she had *“no expectations really”* for birth and not *“too many expectations of myself or [baby] when it comes to routine. I don't force anything”.* At 12 months, Ana reflected on how she took challenges and uncertainties in her stride, without expectations: *“I feel like I went in with very few expectations. Um. I have been lucky to like, I have spent a lot of time with kids in my life and they’re all just so different. That it’s been a little adventure to see, like who this little person is, and to figure out what we like to do together”.*

***Relationship with partner***

**2.a. Developing a great partnership**

Two participants described how they went from snapping at each other due to exhaustion after birth to later developing a great partnership. At one month, Clara explained that *“at the beginning when we were so exhausted there was a couple of snapping at each other back and forth when you know he has to do a lot more 'cause I used to be the one who does all the chores”.* Hope also described occasional snapping at each other when they were tired and a sense that *“we don't see as much of each other as we used to”.* However, at six months, Clara enjoyed taking a lead in parenting, with her partner *“completely implementing it to his parenting style, which I see that he picked up from me and I love it.”* Hope developed a more balanced division of work with partner and a strong sense of togetherness. At six months, they were *“quite good at balancing out jobs and stuff like that, so we've kind of got a bit of a good routine”* and, at 12 months, they were spending *“a lot of time still together”.*

**2.b. Diminished partner support**

Six (out of nine) participants described support from their partner being diminished over time in the first year. Over time, three participants developed growing resentment and frustration about the imbalanced division of parenting. Eve and Ivy described their partners as *“quite supportive”* during pregnancy, however over time this changed. At six months, Ivy explained that she was *“feeling like I was doing a lot. You know my husband getting to go to work and have adult conversations”* and, Ana, who felt unsupported since her pregnancy, felt like she was *“putting all my time and effort into our baby and not my job”.* At 12 months, Ana explained that it came as a *“shock”* that when the baby was born *“overnight you have an entirely new set of priorities”* but this was not the case for her husband. Eve also felt that she did “*a lot more of the childcare bits, and there are sometimes where I felt a bit resentful about that”.*

Three other participants described feeling increasingly alone and distant from their partners. At 1 month, Faye felt that her “*experience with him was just a complete pillar of strength” and* Bria described her husband as *“brilliant”* and them “*working together as well as we normally do”.* However, at six months she explained that *“because he's been so preoccupied with his own stuff around work, I felt like he really withdrew from the marriage emotionally”* and, similarly, Dara felt she and her partner were “*a bit like distant”.* At 12 months, Dara said that she and her partner did not “*spend so much time together*” and Bria felt that she and baby were “*just us on our own”.*

**2.c. Baby receives all the attention and affection**

One participant described the transition from a strong, supportive relationship prenatally to a platonic one later in the first year, with baby receiving all of her attention and affection. Joy described her partner during birth as a *“huge help”* and *“incredibly supportive”,* saying that she was *“really sad that he couldn't stay after the birth* [due to COVID-19 regulation*s]”.* However, at 12 months, she described a more platonic relationship with *“a lot less time for ourselves”* and a partner who *“doesn't get any hugs or kisses anymore [laughs], because baby gets all of that. So that's definitely affection-wise like it does change a lot...”.*

***Socialization***

**3.a. Valuing socialization throughout**

Three participants explained that sharing their pregnancy news with friends and family was important since the beginning. For instance, Ana said that *“telling people the news at that point was really nice because you get all these stories from colleagues who you never talk about personal things (…) and it's nice to observe how people interact with you differently”.* On the other hand, five participants chose to be more private about their pregnancy, keeping it “*our own little secret”* [Bria] and “*being able to be vulnerable in my own space”* [Ivy]. Despite the mixed views of socialization during pregnancy, by 1 month, all of them came to value own and baby’s socialization with family and friends, a feeling that persisted throughout the first year. Clara, for example, made sure that they *“spend a lot of time with friends and family because I am really conscious of making time for her and for the adults to spend time with the baby because I know that they love her”*.

The value of new friendships and contact made with other parents and babies was also highlighted by three participants at different stages of early parenthood. For example, connecting with other parents meant that *“everyone very generously shares information and assistance”* [Ana, 1 month] and having a WhatsApp group made possible that “*you've got somebody to message at like 2 in the morning when you wake up and you feel quite alone”* [Hope, 6 months].

Two participants expressed feelings of nostalgia for their pre-baby social life and support. Joy, at one month, felt they *“had a lot of friends back in the day, a lot of them have now moved away (…) I do feel quite isolated sometimes”.* At 12 months, the struggle was the same, although more focused on not having friends who are parents.

**3.b. Ambivalent about socialization**

One participant felt ambivalent about socialization from pregnancy and as time went on, she wanted to spend more time alone with baby. Faye explained that “*it's really nice to be observed and to be seen pregnant… for friends and family to want to feel it. And occasionally it's not nice (...) people decide that now is a good time to tell you all of their horror stories [laughter] about parenting or about labour, uhm, or about the first few weeks”*. At six months, she said that her *“favorite days with her are when we've got nothing planned.”* At 12 months, she explained that she “*had had a brilliant life of freedom… (now) I wanna be at home, in case my baby needs me”.*

Two more participants, who overall valued socialization, expressed some concerns too. Gail, who is a single mother, explained that sometimes she found social interaction challenging: *“when people hear that I do it on my own, they're like, ohh, is that really hard and they immediately put like the negative on it.”* Hope expressed ambivalence about online support available to parents, such as Facebook support groups, because while this is *“really helpful, like it normalizes a lot and there are some really good advice on these, I think sometimes it can almost be a bit like too doom and gloom”.*

***Coping mechanisms***

**4.a. Anticipating difficulties and taking them in their stride**

Four participants anticipated parenthood difficulties during pregnancy but felt able to cope with challenges ahead. For example, Ana worried about *“life just changing into something that is so unrecognizable”* and Ivy about *“being not a good mother”*. However, when they experienced feeding difficulties in the first month they managed. Ana illustrated the complex process of reflection and considering difficulties as part of parenthood,

*[baby’s] digestion has been off, and so automatically I ascribe it to my decision to try breastfeeding. Whereas it could simply be, the formula. And we have no idea… And I’m trying to keep a distance from it. You’re so in it. But it’s hard to rationally evaluate your behaviors sometimes. So, we second guess ourselves, but then persist, because we must.*

By 12 months, they all felt parenthood was going well. Hope said, *“I didn't know I was capable of loving somebody so much as I do”.* Ana found her previous fears of not returning to work easier to accept when the time came, *“that’s the reality”.* Ivy said, *“Yeah, there's a lot of frustrations and, you know, you do have to channel in to try and stay, stay calm as, as best you can”.*

**4.b. Anticipating and planning but struggling with ongoing challenges**

One mother, Dara, thought ahead of possible difficulties and began planning. The focus during pregnancy *“was about my job and my career… I figured it out.”* At one month, Dara had to change to formula due to baby’s jaundice and felt disappointed. By six months Dara described how the constant changes meant she couldn’t plan as she did before, *“it's hard”.* At 12 months Dara reflects on her previous expectations and approach, *“I thought as they get older, things get easier, but they don't. You get over something and then it's the next challenge”.*

**4.c. Planning but letting go to be more in the moment**

Two participants described planning ahead but later letting go of that to be more in the moment. During pregnancy, Eve tried to plan, *“my anxiety is worse if I don't know what's happening. I like to know what the different options are”.* But after her baby was born, Eve was surprised at how she felt, especially about the difficulty of breastfeeding, *“I hadn't really thought about the impact on how I feel… getting really cross”.* By six months, Eve, with support from a health visitor, decided to stop breastfeeding, *“I just needed someone to say you can't do both”.* It helped hearing someone take her feelings into account. By 12 months she found the different challenges easier to manage.

When pregnant, Faye prepared for the exhaustion of upcoming parenthood by, *“practicing with just being tired but staying calm”.* After having a good experience of soothing her baby at one month, Faye stopped planning ahead, *“I don't really know anything about teeth[ing], but we are nowhere near that stage. I just now just feel confident that I'll figure it out when we get there”.* By 6 months, Faye felt it was more important to listen to her baby than what she had planned or heard “*what other people are doing*”.

**4.d. From God, to others, to self**

One participant, Bria, relied on her faith in God during her pregnancy, *“I've had a real sense of just trust in God and that it is in control. So, I'm not actually fearful.”* After having a difficult birth, Bria’s sense of support extended to people in her community, *“We knew that people were praying for us. We knew people were aware that it wasn't going as smoothly as might have been hoped… I just remember feeling calm.”* The difficulties continued as baby had a medical condition and much need of support, *“It's been quite a learning curve… So, and we've learned a lot quite quickly. I think it's been alright. [to baby] It's most different fun? We seem to be keeping you in one piece, don't we?”* At 12 months, Bria continued with her strong faith and valuing support from others, but she also described a better awareness of her role in her baby’s life, *“At some point, we'll be doing something, and she will need us and it's just like, ‘oh, okay, that's just normal’”.*

**4.e. Avoiding thinking, minimizing, and “getting on with it”**

One participant, Gail, initially managed to have a positive outlook by avoiding thinking about aspects she could not control or were uncertain. As a single parent, Gail’s biggest worry about the future when pregnant was that her baby would not be content with only parent, but she said, *“I don’t have to worry about that now, time will tell”.* Later, when baby was one month and constantly waking up at night, Gail seemed to minimize her sleepless nights, *“I don't worry about that so much because we've got nowhere to be. It’s not the end of the world”.*

By 6 months, Gail seemed to acknowledge the difficulties more, but these felt manageable, *“there are days that are harder than others, but I've never felt like it's so hard I can't cope”.* Similarly, at 12 months, Gail explains how she finds parenthood, *“I think I just get on with it”.*

**4.f. Avoiding thinking and becoming overwhelmed with difficulties**

Another participant, Clara, coped with difficulties during pregnancy by denying or forgetting challenges, *“I can't remember what was what made it challenging, uhm? See, this is the type of person I am I... even if negative things happen, I forget about them.”* During birth she described thinking, *“So this is how I was coping with the whole thing, that it will soon be over… this is just temporary, but I didn't have many other thoughts during labor”.*

At six months, Clara felt she did not have many challenges. Yet, by 12 months, Clara struggled with baby becoming ill and felt she had no sense of control or way to face these difficulties, *“When your baby is unwell, and you can't do anything to make them feel better. It is horrible. Like, I can't even describe how horrible it made me feel seeing her so unwell … no control over this… She was refusing breastfeeding and that was almost like a personal failure to me. That my one thing that always works, just wasn't working for her. So, that was really tough”.*
